# The role of skin testing, drug challenge and IFN-γ ELISpot in delayed hypersensitivity to iodinated contrast media

**DOI:** 10.1186/s13223-025-00982-3

**Published:** 2025-08-14

**Authors:** Ana Maria Copaescu, Kyra Y. L. Chua, Effie Mouhtouris, Natasha E. Holmes, Moneerah AlGassim, Ibtihal Al Otaibi, Florian Stehlin, Ghislaine A. C. Isabwe, Christos Tsoukas, Jean-Francois Toupin, Derek Lee, Moshe Ben-Shoshan, Elizabeth J. Phillips, Jason A. Trubiano

**Affiliations:** 1 Centre for Antibiotic Allergy and Research, Department of Infectious Diseases, Austin Health, Heidelberg, VIC Australia; 2 Department of Medicine, Division of Allergy and Clinical Immunology, McGill University Health Centre (MUHC), McGill University, Montreal, QC Canada; 3 The Research Institute of the McGill University Health Centre, McGill University, McGill University Health Centre (MUHC), Montreal, QC Canada; 4 Department of Infectious Diseases, University of Melbourne, at the Peter Doherty Institute for Infection and Immunity, Melbourne, VIC Australia; 5 Division of Allergy and Clinical Immunology Department of Pediatrics, Central Second Health Cluster, Ministry of Health, Riyadh, Saudi Arabia; 6 Division of Clinical Immunology and Allergy, Department of Medicine, Centre Hospitalier Universitaire Vaudois (CHUV), Lausanne, Switzerland; 7 Department of Diagnostic Radiology, McGill University Health Centre (MUHC), McGill University, Montreal, QC Canada; 8 Pharmacy Department, Montreal General Hospital, Montreal, QC Canada; 9 Division of Allergy, Immunology and Dermatology, Montreal Children’s Hospital, McGill University Health Centre (MUHC), McGill University, Montreal, QC Canada; 10 Institute for Immunology and Infectious Diseases, Murdoch University, Murdoch, WA Australia; 11 Center for Drug Safety and Immunology, Department of Medicine, Dermatology, Pathology, Microbiology & Immunology, Vanderbilt University Medical Centre, Nashville, TN USA; 12 The National Centre for Infections in Cancer, Peter MacCallum Cancer Centre, Parkville, VIC Australia; 13 Montreal General Hospital, 1650 Cedar Ave, Montreal, QC H3G 1A4 Canada

**Keywords:** Delayed hypersensitivity reactions, Maculopapular exanthema, Drug reaction with eosinophilia and systemic symptoms, Iodinated contrast media, In vivo diagnostic tools, Ex vivo diagnostic tools

## Abstract

**Background:**

The use of in vivo and ex vivo diagnostic tools for delayed hypersensitivity reactions (DHRs) associated with iodinated contrast media (ICM) is currently ill-defined.

**Objective:**

To evaluate the role of in vivo and ex vivo diagnostic tools for DHRs occurring >6 h following intravenous low-osmolality ICM.

**Methods:**

We conducted a prospective, multicenter, international cohort study. The patients were recruited from two tertiary care adult allergy clinics, Austin Health, Australia and the McGill University Health Centre, Canada. Eligible participants were adults who reported a DHR after receiving ICM. In vivo testing (skin testing and intravenous challenge) was performed to identify an alternative agent. Ex vivo testing using interferon-γ enzyme-linked ImmunoSpot assay was performed in four Australian patients to explore its diagnostic performance.

**Results:**

The culprit ICM was identified by dIDT in 17/20 (85%) while in 3/20 (15%) a challenge was necessary to confirm delayed hypersensitivity. All patients with a positive dIDT to iohexol were positive to iodixanol (15/15; 100%) while 3/4 (75%), 3/4 (75%), 4/6 (67%), and 3/5 (60%) were positive to iopromide, ioversol, iopamidol, and iobitridol, respectively. Overall, 7/20 (35%) patients tolerated a challenge with an alternative ICM. The IFN-γ release assay was negative for the implicated ICM in 4 patients with confirmed DHR through a positive dIDT.

**Conclusion:**

dIDT allowed confirmation of T cell-mediated allergy to the implicated ICM in 85% of patients with a strong clinical suspicion of DHR and identification of non-cross-reactive ICM in 35% of patients. The IFN-y ELISpot was not useful in the four patients tested.

## Introduction

Iodinated contrast media (ICM) are essential diagnostic agents in modern imaging, yet their use is occasionally complicated by hypersensitivity reactions. These reactions are broadly categorized into immediate (within one hour) and nonimmediate or delayed hypersensitivity reactions (DHRs), which occur from one hour up to 48 h post-exposure [1]. Delayed reactions are predominantly cutaneous, manifesting as benign maculopapular exanthems (MPE) but also severe drug reactions with eosinophilia and systemic symptoms (DRESS), and are believed to be T-cell mediated. DHRs to intravenous low-osmolality ICM comprise an estimated 0.5–23% of all hypersensitivity reactions to contrast material [2]. Despite their frequency, the pathophysiology of DHRs remains incompletely understood, and diagnostic approaches are still evolving. In vivo delayed intradermal testing (dIDT) has been described for these delayed ICM reactions; however, the sensitivity, specificity, positive and negative predictive values are ill-defined [3, 4]. Studies have shown that dIDT using a 1:10 dilution of the culprit agent can help differentiate between allergic and nonallergic reactions and guide the selection of alternative agents [5]. However, a well-known position paper publication has used full-strength concentrations for dIDT to maximize sensitivity, particularly in cases of non-immediate hypersensitivity [6].

Various iodinated ICM are available in Canada and Australia, including nonionic monomers such as iohexol and nonionic dimers like iodixanol (Table 1). Structural similarities among these agents are clinically significant, as cross-reactivity is common [3–5]. For instance, iodixanol is a dimer of iohexol, and patients sensitized to one may react to the other due to shared molecular epitopes [5].

**Table 1:** Available iodinated contrast media used in this study

Name	Commercial name	Common indication	Half life	ELISpot concentrations (μg/ml)
Iohexol	Omnipaque ®	Computed tomography, coronary angiography	5–10 min	200–500–2000
Iodixanol	Visipaque ®	Coronary angiography	2.1 h	200–500–2000
Iopromide^a^	Ultravist ®	Intravenous procedures Computed tomography	2 h	500–1000
Ioversol^a^	Optiray ®	Intravenous procedures	5–10 min	200–500–2000
Iobitridol^a^	Xenetix ®	Intravenous procedures Computed tomography	1.8 h	n/a
Iopamidol^b^	Isovue ®	Computed tomography	2 h	n/a

^a^These agents were tested only in the Australian cohort

^b^This agent was only tested in the Canadian cohort

*LISpot* enzyme-linked ImmunoSpot assay, *n/a* not applicable

Ex vivo testing, such as lymphocyte transformation tests (LTT), has also been explored, though its clinical utility remains limited due to variability in sensitivity and specificity [7–9]. Nonetheless, these tests may offer additional insights in complex cases or when skin testing is contraindicated. To our knowledge, interferon-γ (IFN-γ) enzyme-linked ImmunoSpot assay (ELISpot) has never been performed using these agents.

This exploratory study aims to evaluate the role of in vivo and ex vivo diagnostic tools for DHRs occurring > 6 h following intravenous low-osmolality ICM.

## Methods

Patients with ICM-associated well-phenotyped MPE, DRESS, and acute generalized exanthematous pustulosis (AGEP) were prospectively recruited from two tertiary care adult allergy clinics: Austin Health, Australia, and McGill University Health Centre, Canada.

In vivo intradermal testing with undiluted and tenfold diluted ICM was performed with the implicated agents (Naranjo score ≥ 5) and available ICM alternatives (Table 1). Iohexol and iodixanol were used for testing in both centers (Table 1). Additional testing was performed in Australia with iopromide, ioversol and iobitridol, depending on availability. However, in Canada, iopamidol was the third agent used for testing (Table 1). For each patient, the implicated ICM agents were confirmed with the respective radiology departments (Table 2). Delayed IDT (injected volume of 0.02–0.05 ml) was performed at least 6 weeks after the ICM-associated reaction, as recent recommendations regarding safety testing suggested [10]. Delayed IDT was assessed through photographic evidence submitted by the patient via secure email or text message to the treating team and by patient-reported symptoms at 24 and 48 h. The treating team maintained consistency in assessment by ensuring that the photographic submissions and symptom reports were evaluated within this timeframe. Patients were asked explicitly whether the lesion was palpable or blistered to minimize misclassification bias and aid in distinguishing induration from erythema. A positive dIDT was defined as a persistent erythematous induration at the injection site between 6 h and up to 48 h. The ICM concentrations appeared non-irritant following delayed testing on 5 healthy controls from Australia and 5 from Canada. Following dIDT, an ICM intravenous challenge consisting of one 15 ml dose was performed to identify a suitable alternative contrast agent (Table 2).

**Table 2:** In vivo testing results*

ID	Sex/age	CCI/PMH	Culprit agent	ICM reactions	Time since last reaction (days)^a^	Delayed IDT –	ICM challenge	Tolerated subsequent ICM CT-scan^b^
**Patient 1** AUS-SCAR	F 86	CCI:5 AF, HTN, CKD	Iohexol (Omnipaque ®)	2 non-severe MPE RegiSCAR-1 (12/2016) RegiSCAR 0 (04/2020) 1 DRESS RegiSCAR 5 (05/2020)	177	**Positive** Iohexol 1/10 and 1/1 Iodixanol 1/10 and 1/1 Iopromide 1/10 and 1/1 Ioversol 1/10 and 1/1	None	No
						Negative None		
**Patient 2** PIPA	M 66	CCI:4 Ischemic CM, CHD	Iohexol (Omnipaque ®)	2 non-severe MPE RegiSCAR-2 (06/2009) RegiSCAR-1 (04/2020)	208	**Positive** Iohexol 1/10 and 1/1 Iodixanol 1/10	**Negative ioversol IV challenge**	No
						Negative Iopromide 1/10 and 1/1 Ioversol 1/10 and 1/1		
**Patient 3** PIPA	M 34	CCI: 3 Acute promyelocytic Leukemia, DM2, Bipolar disorder	Iohexol (Omnipaque ®)	1 DRESS^c^ RegiSCAR 6 (02/2020) 1 severe MPE^d^ RegiSCAR 2 (09/2020)	65	**Positive** Iohexol 1/10 and 1/1 Iodixanol 1/10 and 1/1 Iopromide 1/1 Ioversol 1/10 and 1/1 Iobitridol 1/10 and 1/1 (Blistering reactions at the injection site—Fig. 2A)	None	No
						Negative None		
**Patient 4** PIPA	F 62	CCI: 6 ESRD—IgA nephropathy, Langerhans cell histiocytosis	Iohexol (Omnipaque ®)	3 non-severe MPE RegiSCAR-2 (09/2018) RegiSCAR-2 (05/2019) RegiSCAR-2 (09/2020)	65	**Positive** Iohexol 1/1 Iodixanol 1/1 **Equivocal** Iopromide 1/1 Ioversol 1/1	**Positive ioversol IV challenge** (15 ml)—MPE (requiring Prednisolone 50 mg x 3d) **Negative iobitridol IV challenge**	**Yes** **(Iobitridol)**
						Negative Iobitridol 1/10 and 1/1		
**Patient 5** PIPA	M 68	CCI:3 MS, AF, DM2	Iohexol (Omnipaque ®)	2 non-severe MPE RegiSCAR-1 (08/2021) RegiSCAR 1 (03/2023)	177	**Positive** Iohexol 1/10 and 1/1 Iodixanol 1/10 and 1/1	**Negative iobitridol IV challenge**	**Yes** **(Iobitridol)**
						Negative Iobitridol 1/10 and 1/1		
**Patient 6** PIPA	F 61	CCI:6 ESRD – HTN, Lymph node TB, Bronchiectasis	Iohexol (Omnipaque ®) Iodixanol (Visipaque®)	1 severe MPE^d^—iohexol RegiSCAR 2 (09/2019) 1 non-severe MPE—iodixanol RegiSCAR -2 (10/2020)	188	Positive None	**Positive iohexol IV challenge** (15 ml)—MPE (requiring Prednisolone 50 mg × 5d) **Negative iobitridol IV challenge**	No
						Negative Iohexol 1/10 and 1/1 Iodixanol 1/10 and 1/1 Iopromide 1/10 and 1/1 Ioversol 1/10 and 1/1		
**Patient 7** IDEAL	M 34	CCI: 1 Congenital biliary atresia (liver transplant)	Iohexol (Omnipaque ®)	2 non-severe MPE RegiSCAR -2 (2009 and 2010)	4,015	**Positive** Iohexol 1/10 and 1/1 Iodixanol 1/10 and 1/1 Iopamidol 1/10 and 1/1	None	No
						Negative None		
**Patient 8** IDEAL	M 75	CCI: 5 Parkinson’s disease, HTN, CHD DRESS (2018) to Meropenem (confirmed by dIDT) and Vancomycin (HLA-A:32:01)^d^	Iohexol (Omnipaque ®) Iodixanol (Visipaque ®)	2 non-severe MPE RegiSCAR -2 (1 episode with each product) (12/2021 and 2020)	270	**Positive** Iohexol 1/10 and 1/1 Iodixanol 1/10 and 1/1 Iopamidol 1/10 and 1/1 (Fig. 2B)	None	No
						Negative None		
**Patient 9** IDEAL	F 62	CCI: 2 No PMH	Iodixanol (Visipaque ®)	1 non-severe MPE RegiSCAR-3 (02/2019)	1245	**Positive** Iodixanol 1/10 and 1/1	None (refused)	No
						Negative Iohexol 1/10 and 1/1 Iopamidol 1/10 and 1/1		
**Patient 10** PIPA	M 64	CCI:3 DM2, HTN, Necrotizing pancreatitis Vancomycin DRESS (10/2019)^f^	Iohexol (Omnipaque ®)	1 severe MPE^d^ RegiSCAR 2 (08/2022)	71	**Positive** Iohexol 1/10 and 1/1 Iodixanol 1/10 and 1/1 Iobitridol 1/10 and 1/1 (Fig. 2.E. and F.)	None	No
						Negative None		
**Patient 11** IDEAL	M 52	CCI: 3 Hairy cell leukemia Ampicillin DRESS (01/2020)	Iohexol (Omnipaque ®)	3 non-severe MPE RegiSCAR-3 (02/2020; 05/2020 12/2020)	700	**Positive** Iohexol 1/10 and 1/1 Iodixanol 1/10 and 1/1	**Negative iopamidol challenge**	No
						Negative Iopamidol 1/10 and 1/1		
**Patient 12** IDEAL	M 69	CCI: 3 CHD	Iodixanol (Visipaque ®)	1 non-severe MPE RegiSCAR 0 (11/2021)	485	**Positive** Iodixanol 1/10 and 1/1	**Negative iopamidol challenge** **Negative iohexol challenge**	**Yes (Iopamidol)**
						Negative Iohexol 1/10 and 1/1 Iopamidol 1/10 and 1/1		
**Patient 13** IDEAL	F 64	CCI: 2 HTN, pre-DM2, OA	Iohexol (Omnipaque ®)	AGEP EuroSCAR 8 (12/2021)	455	**Positive** Iohexol 1/10 and 1/1 Iodixanol 1/1 (Fig. 2C)	**Negative iopamidol challenge**	No
						Negative Iopamidol 1/10 and 1/1		
**Patient 14** IDEAL	F 43	CCI: 0 Spondyloarthritis (HLA-B27 +)	Iohexol (Omnipaque ®)	2 non-severe MPE RegiSCAR 0 (04/2022)	335	**Positive** Iohexol 1/10 and 1/1 Iodixanol 1/10 and 1/1 Iopamidol 1/10 and 1/1 (Fig. 2D)	None	No
						Negative None		
**Patient 15** IDEAL	F 32	CCI: 0 SLE (nephritis), HypoT4 Ertapenem vs Vancomycin AGEP EuroSCAR 3 (08/2021)	Iohexol (Omnipaque ®)	2 non-severe MPE RegiSCAR-3 (04/2022; 09/2021)	90	Positive None *Pending repeat dIDT*	**Positive iohexol IV challenge** (15 ml)—MPE (no treatment) (Fig. 2I)	No
						Negative Iohexol 1/10 and 1/1 Iodixanol 1/10 and 1/1 Iopamidol 1/10 and 1/1		
**Patient 16** IDEAL	M 62	CCI: 2 DSLP, Gout, Psoriasis	Iohexol (Omnipaque ®)	1 non-severe MPE RegiSCAR-3 (12/2020)	575	Positive None *Pending repeat dIDT*	**Positive iohexol IV challenge** (15 ml)—MPE (no treatment) (Fig. 2H)	No
						Negative Iohexol 1/10 and 1/1 Iodixanol 1/10 and 1/1 Iopamidol 1/10 and 1/1		
**Patient 17** IDEAL	F 78	CCI: 7 CLL, hypogammaglobulinemia, DM1, HypoT4	Iohexol (Omnipaque ®)	1 non-severe MPE RegiSCAR 1 (06/2020)	1426	**Positive** Iohexol 1/10 and 1/1 Iodixanol 1/10 and 1/1 Iopamidol 1/10 and 1/1	None	No
						Negative None		
**Patient 18** PIPA	F 54	CCI: 1 HypoT4, PVT, bariatric surgery Tazocin DRESS/mini-DRESS (12/2019)	Iohexol (Omnipaque ®)	1 non-severe MPE RegiSCAR-2 (05/2023)		**Positive** Iohexol 1/10 and 1/1 Iodixanol 1/10 and 1/1 Iobitridol 1/10 and 1/1	None	No
						Negative None		
**Patient 19**	M 48	CCI: 1 MV repair due to endocarditis	Iohexol (Omnipaque ®)	2 severe MPE RegiSCAR 2 (08/2022)	783	**Positive** Iohexol 1/10 and 1/1 Iodixanol 1/10 and 1/1	**Positive iobitridol IV challenge** (15 mL)—localized reaction at site of previous iobitridol dIDT testing (Fig. 2G)	No
						Negative Iobitridol 1/10 and 1/1		
**Patient 20**	F 70	CCI: 5 Small bowel neuroendocrine tumor, HTN, OA	Iohexol (Omnipaque ®)	1 non-severe MPE Regiscar-1	140	**Positive** Iohexol 1/10 and 1/1 Iodixanol 1/10 and 1/1	None	No
						None		

^a^When several reactions were reported, we indicated the time since the most recent reaction. This delay represents the latency between the reaction and the initial allergy investigations

^b^The tolerated subsequent ICM during CT-scan refers to patients who tolerated alternative ICM following their initial reaction. The follow-up was performed until December 2024

^c^Patient #3 had a concurrent antibiotic allergy with a positive dIDT to piperacillin/tazobactam and clavulanic acid. These two antibiotics were also considered culprits for the DRESS

^d^Severe MPE was defined as an extensive cutaneous exanthem with more than 50% of body surface area and RegiSCAR score of 2 to 3 (possible), as previously defined (Ref: Trubiano JA, Chua KYL, Holmes NE, et al. Safety of cephalosporins in penicillin class severe delayed hypersensitivity reactions. J Allergy Clin Immunol Pract 2020; 8(3): 1142-6 e4)

^e^Patient #8 had a history of DRESS in 2018 with culprits that included meropenem (dIDT positive) and vancomycin (positive HLA-A32:01)

^f^Patient #10 has a past medical history of Vancomycin -DRESS confirmed by both positive dIDT and ex vivo ELISpot

*AF* atrial fibrillation, *AGEP* acute generalized exanthematous pustulosis, *CCI* Charlson Comorbidity Index, *CHD* coronary heart disease, *CLL* chronic lymphocytic leukemia, *CMP* cardiomyopathy, *CKD* chronic kidney disease, *CT* computed tomography, *dIDT* delayed reading intradermal testing, *DM1* diabetes myelitis type 1, *DM2* diabetes myelitis type 2, *DRESS* drug reaction with eosinophilia and systemic symptoms, *DSLP* dyslipidemia, *ESRD* end-stage renal disease, *EuroSCAR* standardized AGEP scoring system, *HTN* hypertension, *HypoT4* hypothyroidism, *ICM* iodinated contrast media, *IV* intravenous, *L* left, *MI* myocardial infarct, *MPE* maculopapular exanthema, *MS* multiple sclerosis, *OA* osteoarthritis, *SLE* systemic lupus erythematosus, *TB* tuberculosis

**Cohorts**:

PIPA Study, Predictors, Immunopathogenesis and Prescribing in Antibiotic allergy, Australia

AUS-SCAR Study, AUStralian registry of Severe Cutaneous Adverse Reactions, Australia

IDEAL Study, DIagnostic anD predictor Tools for ImmunE-mediated Drug Allergy, Canada

*Note*: Culprit contrast agents were confirmed with the radiology department from each institution

*Ex vivo testing* was performed with the patient’s peripheral blood mononuclear cells (PBMC) stimulated with the relevant non-cytotoxic ICM concentrations for T cell dose-dependent IFN-γ release. An ELISpot assay was used, as per previous publications [11]. The IFN-γ ELISpot assay was performed using a negative (unstimulated, media alone) and a positive control (anti-human CD3 antibody, 1-D1K, Mabtech®). Iodixanol, iohexol, ioversol and iopromide (Sapphire Biosciences) were used in a subgroup of 4 patients. These patients were selected based on previous positive dIDT and consent to provide blood samples for this specialized assay. The non-cytotoxic concentration ranges utilized in the assay were determined using the LDH-Cytotoxicity Assay Kit II as per the manufacturer’s instructions (LDH Assay, Abcam). The average number of spots for the test and unstimulated wells was calculated. As per previously published definitions, a positive response was defined as equal to or greater than 50 spot-forming units (SFU)/million cells after background (unstimulated control) removal [12, 13]. Spots were analyzed automatically with an AID ELISpot Reader (software version 7.0; Mabtech®).

## Results

**Fig. 1:**
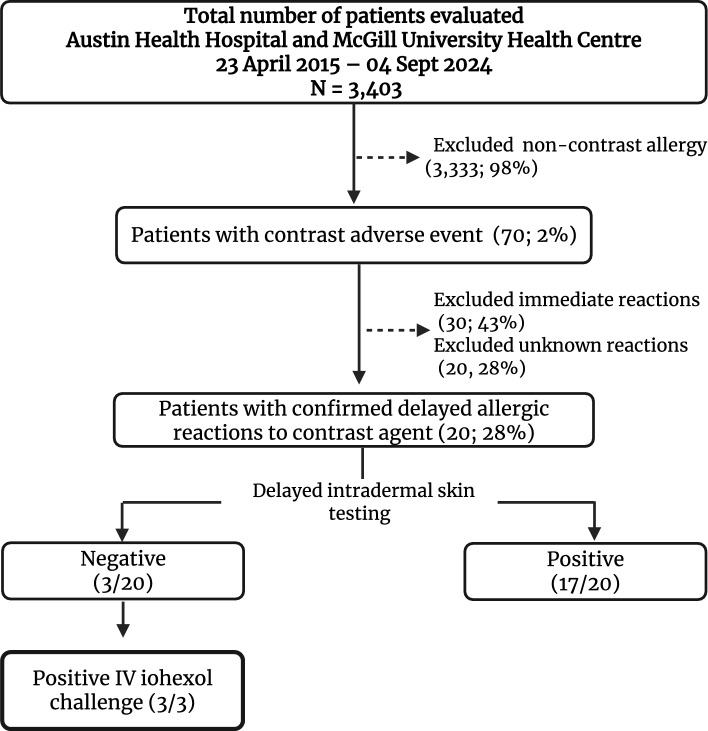
Participant flow diagram

Among the 3403 patients evaluated for a drug allergy label in both centers, 70 reported a history of contrast adverse reaction (Fig. 1). Among these, 20 patients with 36 radiocontrast-associated DHRs were enrolled (Table 2), including 10 from Australia and 10 from Canada, between 2015 and 2024. Reactions occurred in patients who had not undergone specific premedication protocols to prevent them, with some individuals also having a history of multiple reactions. Iohexol alone was implicated in 16 subjects, iodixanol alone was involved in 2 subjects, and 2 subjects reacted after both iohexol and iodixanol.

**Fig. 2:**
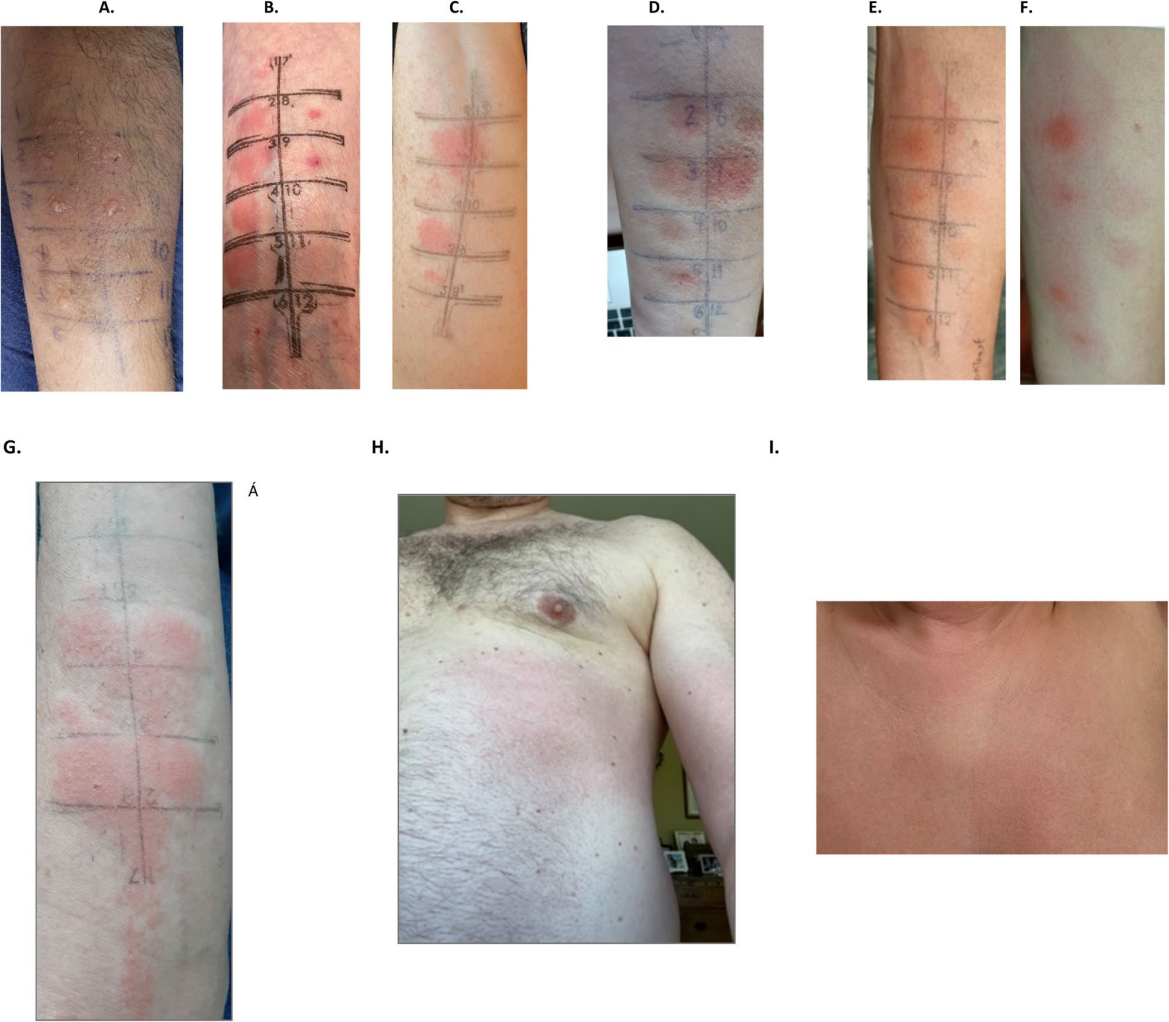
Examples of delayed positive intradermal skin testing and intravenous challenges. **A** *Patient 3*—Positive for iohexol 1/10 (#2) and 1/1 (#8), iodixanol 1/10 (#3) and 1/1 (#9), iopromide 1/10 (#4) and 1/1 (#10), and ioversol 1/10 (#5) and 1/1 (#11). This patient had no systemic reaction following IDT and required topical and oral corticosteroids (prednisolone 25 mg PO for 3 days). **B** *Patient 8*—Positive for iohexol 1/10 (#2) and 1/1 (#3), iodixanol 1/10 (#4) and 1/1 (#5), iopamidol 1/10 (#8) and 1/1 (#9) at 12 h after intradermal testing. **C** *Patient 13*—Positive for iohexol 1/10 (#2) and 1/1 (#3), and iodixanol 1/1 (#5) on day 3 after intradermal testing. **D** *Patient 14*—Positive for iohexol 1/10 (#2) and 1/1 (#3), iodixanol 1/10 (#4) and 1/1 (#5), iopamidol 1/10 (#8) and 1/1 (#9) at 24 h after intradermal testing. **E** *Patient 10*—Positive for iohexol 1/10 (#2) and 1/1 (#3), iodixanol 1/10 (#5) and 1/1 (#6), and iobitridol 1/10 (#9) and 1/1 (#10). This patient had no systemic reaction following IDT and required topical corticosteroids. **F** *Patient 10*—Delayed IDT after 1 week. **G** *Patient 19*—Positive non-severe delayed reaction following IV challenge with iobitridol, presenting as a localized skin eruption at the previous IDT site for iohexol, iodixanol, and iobitridol. **H** *Patient 16*—Positive non-severe delayed reaction following IV challenge with iohexol, occurring 48 h after the challenge and lasting 72 h. No treatment was required. **I** *Patient 15*—Positive non-severe delayed reaction following IV challenge with iohexol

*Delayed intradermal testing* All 20 patients underwent dIDT, with 17 testing positive and 3 testing negative (Fig. 1). The three patients with negative dIDT (patients 6, 15 and 16) underwent an IV challenge with iohexol, and all had a positive non-severe IV challenge (no premedication) (Figs. 1, 2).

*ICM cross-reactivity*. Significant cross-reactivity was observed between iodixanol (the dimer of iohexol) and iohexol (Fig. 1). Among the 15 patients with a history of reaction to iohexol and a positive dIDT to iohexol, 100% (15/15) had a positive dIDT to iodixanol. Inversely, among the two patients with a history of reaction to iodixanol, both tested positive only to iodixanol on dIDT and one accepted iohexol and iopamidol challenges and tolerated them (Table 3).

**Table 3:** ICM hypersensitivity testing results and cross-reactivity

Patient ID	Curprit ICM	dIDT Iohexol	dIDT Iodixanol	dIDT Iopromide	dIDT Ioversol	dIDT Iobitridol	dIDT Iopamidol	Challenge ICM	Challenge outcome
1	Iohexol	P	P	P	P	n/a	n/a	n/a	n/a
2	Iohexol	P	P	N	N	n/a	n/a	Ioversol	N
3	Iohexol	P	P	P	P	P	n/a	n/a	n/a
4	Iohexol	P	P	E	E	N	n/a	Ioversol	P
								Iobitridol	N
5	Iohexol	P	P	n/a	n/a	N	n/a	Iobitridol	N
6	Iohexol	N	N	N	N	n/a	n/a	Iohexol	P
								Iobitridol	N
7	Iohexol	P	P	n/a	n/a	n/a	P	n/a	n/a
8	Iodixanol	P	P	n/a	n/a	n/a	P	n/a	n/a
9	Iodixanol	N	P	n/a	n/a	P	N	n/a	n/a
10	Iohexol	P	P	n/a	n/a	N	n/a	n/a	n/a
11	Iohexol	P	P	n/a	n/a	n/a	N	Iopimadol	N
12	Iodixanol	N	P	n/a	n/a	n/a	N	Iohexol	N
								Iopamidol	N
13	Iohexol	P	P	n/a	n/a	n/a	N	Iopamidol	N
14	Iohexol	P	P	n/a	n/a	n/a	P	n/a	n/a
15	Iohexol	N	N	n/a	n/a	n/a	N	Iohexol	P
16	Iohexol	N	N	n/a	n/a	n/a	N	Iohexol	P
17	Iohexol	P	P	n/a	n/a	n/a	P	n/a	n/a
18	Iohexol	P	P	n/a	n/a	P	n/a	n/a	n/a
19	Iohexol	P	P	n/a	n/a	N	n/a	Iobitridol	P
20	Iohexol	P	P	n/a	n/a	n/a	n/a	n/a	n/a

*AF* atrial fibrillation, *AGEP* acute generalized exanthematous pustulosis, *CCI* Charlson Comorbidity Index, *CHD* coronary heart disease, *CLL* chronic lymphocytic leukemia, *CMP* cardiomyopathy, *CKD* chronic kidney disease, *CT* computed tomography, *dIDT* delayed reading intradermal testing, *DM1* diabetes myelitis type 1, *DM2* diabetes myelitis type 2, *DRESS* drug reaction with eosinophilia and systemic symptoms, *DSLP* dyslipidemia, *ESRD* end-stage renal disease, *EuroSCAR* standardized AGEP scoring system, *HTN* hypertension, *HypoT4* hypothyroidism, *ICM* iodinated contrast media, *IV* intravenous, *L* left, *MI* myocardial infarct, *MPE* maculopapular exanthem, *MS* multiple sclerosis, *OA* osteoarthritis, *SLE* systemic lupus erythematosus, *TB* tuberculosis, *dIDT* delayed reading intradermal testing, *E* equivocal, *ICM* iodinated contrast media, *P* positive, *N* negative, *n/a* not applicable

Among the 15 patients with a positive dIDT to iohexol, cross-reactive responses were observed between iohexol and iopromide (3/4, 75%; including one equivocald dIDT result), iohexol and ioversol (3/4, 75%), iohexol and iopamidol (4/6, 67%) and iohexol and iobitridol (3/5, 60%)—(Tables 2 and 3). In addition, patient 6, in whom reactivity to iohexol was confirmed by challenge after a negative dIDT, tolerated an iobitridol challenge leading to a cross-reactivity rate of 50% (3/6). Except for the patient who tested negative to iopromide, tolerance to all other non-cross-reactive ICM was confirmed by challenge. Three patients (1, 3 and 4) had positive dIDT to ioversol, iopromide, iodixanol and iohexol. Patients 3, 10 and 18 had a positive dIDT to iohexol, iodixanol and iobitridol. Four patients (7, 8, 14 and 17) had a positive dIDT to iohexol, iodixanol and iopamidol. Patient 19, who presented with severe maculopapular exanthem associated with iohexol, had positive dIDT to both iohexol and iodixanol and negative dIDT to iobitridol. The patient had no prior exposure to iobitridol. Based on the negative dIDT, an IV challenge with iobitridol was performed, which resulted in a localized skin eruption. This eruption occurred at the previous iobitridol intradermal testing site, which was distinct from the sites used for iohexol and iodixanol testing.

*ICM challenge.* Overall, 7/20 (35%) patients could tolerate a challenge with an alternative ICM (Table 2).

*Ex vivo testing*. The IFN-γ ELISpot was performed in 4 patients across a range of concentrations with inconsistent results in the context of a clear clinical history and positive dIDT (Table 4). In all 4 patients, the assay failed to identify the culprit ICM while showing the highest IFN-γ release to ioversol, including in patient 2, who subsequently tolerated an intravenous challenge with this same agent.

**Table 4:** Ex vivo testing results—ELISpot Results measuring interferon-gamma release by T lymphocytes in response to co-incubation with iodinated contrast media

ID	Culprit agent	Positive dIDT	Time since last reaction (days)^a^	Allergy testing outcome	ELISpot
Patient 1	Iohexol (Omnipaque ®)	Iohexol 1/10 and 1/1 Iodixanol 1/10 and 1/1 Iopromide 1/10 and 1/1 Ioversol 1/10 and 1/1	194	Avoid all ICM	
Patient 2	Iohexol (Omnipaque ®)	Iohexol 1/10 and 1/1 Iodixanol 1/10	271	Negative ioversol IV challenge	
Patient 3	Iohexol (Omnipaque ®)	Iohexol 1/10 and 1/1 Iodixanol 1/10 and 1/1 Iopromide 1/1 Ioversol 1/10 and 1/1	244	Avoid all ICM	
Patient 4	Iohexol (Omnipaque ®)	Iohexol 1/1 Iodixanol 1/1 Equivocal to Iopromide 1/1 Ioversol 1/1	51	Negative iobitridol IV challenge	
Healthy control	n/a	n/a	n/a	n/a	

*dIDT* delayed reading intradermal testing, *SFU* spot-forming units

^a^This delay represents the latency between the reaction and the PBMC collection

The IFN-γ ELISpot assay was performed using a negative (unstimulated, media alone), a positive control (Mabtech anti-human CD3 antibody) and the test contrast agents. A positive response was defined as an average number of 50 or above spot forming units (SFU) per million cells (*red dotted line*) after subtraction of background as per previously published [13]

The 30 SFU/10^6^ cells (*black dotted line*) are also illustrated in the Figures. The *blue dotted line* indicates that the positive responses are greater than the mean of all the background samples plus 2× the standard deviation (SD) of the background [19]

## Discussion

In our cohort, broad cross-reactivity was observed following dIDT for patients with a history of delayed hypersensitivity to ICM, particularly between iohexol and iodixanol. Two patients tolerated iobitridol, a structurally different product, following a positive dIDT for the other available agents. The study also demonstrated that dIDT for ICM has higher-than-expected specificity, dIDT being positive for the culprit ICM in most patients with a well-defined clinical phenotype, while highlighting the possibility of false-negative dIDT results in a minority. It suggests the necessity of conducting IV challenges after a negative dIDT before ruling out a hypersensitivity reaction. Four patients also had a history of antibiotic-associated DRESS/AGEP months to years before their ICM-related reaction, suggesting a possible multiple-drug hypersensitivity syndrome [14].

In this exploratory study, in vivo tools such as skin testing and challenge were more reliable diagnostic solutions than ex vivo testing. These tools were also very safe, with no systemic reaction reported, similar to what was previously described in the literature [15]. The NPV of a negative skin test and challenge has been calculated at 90%-96% in some clinical studies [16]. However, dIDT in patients without a reaction history cannot reliably predict a hypersensitivity reaction [17]. While the negative skin testing in controls suggested low false-positive rates, particularly for delayed reactions, it is also important to note that not all patients with positive dIDT results were challenged with the culprit agent. IFN-γ ELISpot ex vivo testing was not helpful as the ICM concentrations were cytotoxic to the patient’s cells, and our results demonstrated no consistent output.

There are several limitations to this study. There was a selection bias for our cohort as we tested patients who reported recent, sometimes multiple, well-phenotyped reactions and only included patients with positive in vivo testing. In this small cohort, we did not collect data to determine the sensitivity or specificity of these testing methods. Another limitation is that positively skin-tested patients were not challenged with the culprit agent to assess the positive predictive value. Nonetheless, the association of a severe reaction with a positive skin test increases the possibility of a reaction upon re-exposure. Another limitation is the low volume of ICM used for challenge (15 ml) compared to the usual volume range for computed tomography (50–80 ml), which could have led to false negative challenge results.

Given the broad cross-reactivity demonstrated in moderate-severe delayed hypersensitivities and the structural similarity between the radiocontrast agents, clinicians should be cautious of a direct challenge to an alternative agent in the context of a severe delayed reaction. In this setting, an in vivo-based approach may be of clinical benefit to direct safe usage for the included phenotypes. In low-risk ICM-reported reactions, a direct challenge with a structurally unrelated product could be safe and valuable from an economic and resource perspective, such as iopamidol or iobitridol in patients with a history of reaction to iohexol [18]. It is also intriguing that, in our cases, multiple exposures and reactions could have facilitated the durability and broad range of reactivity to different ICMs. In this context, the time since the reaction may expedite the loss of a response to cross-reactive dyes, before the loss of reactivity to the implicated agent, but this requires further examination. It is important to note that not all patients tolerate a full-dose diagnostic dye administration, limiting our understanding of potential dose-dependent sensitization issues. The primary culprit agent may also be slower to lose skin test reactivity over time compared to cross-reactive agents, which may require a re-sensitization step.

In conclusion, in vivo tools like skin testing and challenge tests have proven to be safer and more reliable diagnostic methods compared to ex vivo testing. Utilizing an in vivo-based approach could offer clinical benefits by ensuring the safe use of ICM for patients with DHR.

## Data Availability

No datasets were generated or analysed during the current study.

## Abbreviations

DHR: Drug hypersensitivity reaction
ICM: Iodinated contrast media
dIDT: Delayed reading intradermal testing
MPE: Maculopapular exanthema
DRESS: Drug reaction with eosinophilia and systemic symptoms
AGEP: Acute generalized exanthematous pustulosis
PBMC: Peripheral blood mononuclear cells
IFN-γ: Interferon-γ
ELISpot: Enzyme-linked ImmunoSpot assay
IV: Intravenous
NPV: Negative predictive value
